# Regional Temperature Response in Central Asia to National Committed Emission Reductions

**DOI:** 10.3390/ijerph16152661

**Published:** 2019-07-25

**Authors:** Jintao Zhang, Fang Wang

**Affiliations:** 1 Key Laboratory of Land Surface Pattern and Simulation, Institute of Geographic Sciences and Natural Resources Research, Chinese Academy of Sciences, Beijing 100101, China; 2 College of Resources and Environment, University of Chinese Academy of Sciences, Beijing 100049, China

**Keywords:** INDC pledge, Central Asia, extreme high temperature

## Abstract

National committed greenhouse gas emission reduction actions are the center of the Paris Agreement, and are known as ‘Intended Nationally Determined Contributions’ (INDC) that aim to slow down global warming. The climate response to INDC emission reduction is a focus in climate change science. In this study, data from 32 global climate models from the Coupled Model Intercomparison Project Phase 5 (CMIP5) were applied to investigate the changes in the mean and extreme high temperatures in Central Asia (CA) under the INDC scenario above the present-day level. The results show that the magnitude of warming in CA is remarkably higher than the global mean. Almost all the regions in CA will experience more intense, more frequent, and longer-lasting extreme high-temperature events. In comparison with the INDC scenario, the reduced warming of the 2.0 °C/1.5 °C target scenarios will help avoid approximately 44–61%/65–80% of the increase in extreme temperature events in terms of the intensity, frequency, and duration in CA. These results contribute to an improved understanding of the benefits of limiting global warming to the 2.0 °C/1.5 °C targets, which is paramount for mitigation and adaptation planning.

## 1. Introduction

Extreme high-temperature events have continued to occur as global warming has continued in the last several decades; a significant increase in the number of extreme warm days has been observed on most continents, while the number of extreme cold days has remarkably decreased [1,2]. With the enhancement of global warming, the frequency and intensity of heat waves increase. These natural disasters can lead to illnesses and the deaths of people and animals and great economic losses [3,4].

Central Asia (CA) has a typical temperate continental climate that is characterized by sharp temperature ranges, intensive evaporation, and dry and rainless environments, which depend on the location and topography. As one of the world’s most arid areas, CA has an extremely fragile natural environment and ecological system, and thus is extremely sensitive to climate change [5]. The rate of the temperature increase is much higher than the global rate or that in the Northern Hemisphere [6,7]. Recently, CA has received increasing interest from scientific communities, and many research projects have focused on the environmental and ecological variations in this region [8,9,10,11]. However, information about the variations in climate extremes and their effects on ecological and social systems remains scarce. The extent of the impact of regional warming, which can be avoided by lowering the warming level, deserves special attention. Therefore, it is necessary to understand the changes of high-temperature events in CA under different climate scenarios.

The Paris Agreement set a limit on the global mean surface air temperature (SAT) change of 2.0 °C and an aspirational limit of 1.5 °C relative to preindustrial levels [12,13]. To achieve this goal, countries submitted national mitigation plans in the form of Intended Nationally Determined Contributions (INDC). As of May 2019, a total of 192 countries have reported their respective INDC mitigation targets to the United Nations, accounting for the current mitigation commitments negotiated by governments [14].

Future greenhouse gas (GHG) emissions will have an important impact on regional climate extremes in the next few decades. By considering the INDC emission scenarios and synthesizing national INDC mitigation targets, this bottom–up approach reflecting the willingness of each country to reduce their GHG emissions is more realistic, because it avoids the divergence of different countries from the distribution quota [15]. Nevertheless, few recent studies made use of the INDC scenarios and mainly focused on the global mean temperature responses [16,17,18,19]. Few studies evaluated the possible changes of regional extreme high-temperature events under the INDC pledges, so that the socioeconomic system risks associated with the temperature-related extreme change in the next few decades are still unknown.

In this study, the regional mean and extreme high-temperature changes in CA are examined in association with the INDC emission scenarios and compared with those based on the 2.0 °C/1.5 °C scenarios. The key questions are (a) how will the regional SAT change under the INDC scenario in different regions of CA, (b) how will the extreme high-temperature change under the INDC scenario, and (c) what impacts can be avoided in CA by lowering the warming level (e.g., 2.0 °C or 1.5 °C) compared with the INDC scenario? The multi-model ensemble of 32 global climate models (GCMs) from the Fifth Coupled Climate Model Intercomparison Project (CMIP5) was used in this study.

## 2. Data and Method 

### 2.1. Scenarios

In this study, the INDC mitigation target scenarios based on the Paris Agreement are analyzed. The INDC scenarios are based on Nationally Determined Contributions (NDC) emission data submitted by 192 countries [14]. In this study, the 165 national NDC that were submitted as of May 2019 were analyzed; the European Union member states submitted one NDC target for the whole region. The reported national emission targets range from absolute to relative base year levels or represent emission reduction targets relative to the baseline emission scenarios. We calculated each country’s future quantified emissions targets using the normalization method. For additional information on the NDC dataset, please refer to the study of Wang et al. (2018) [20].

**Figure 1:**
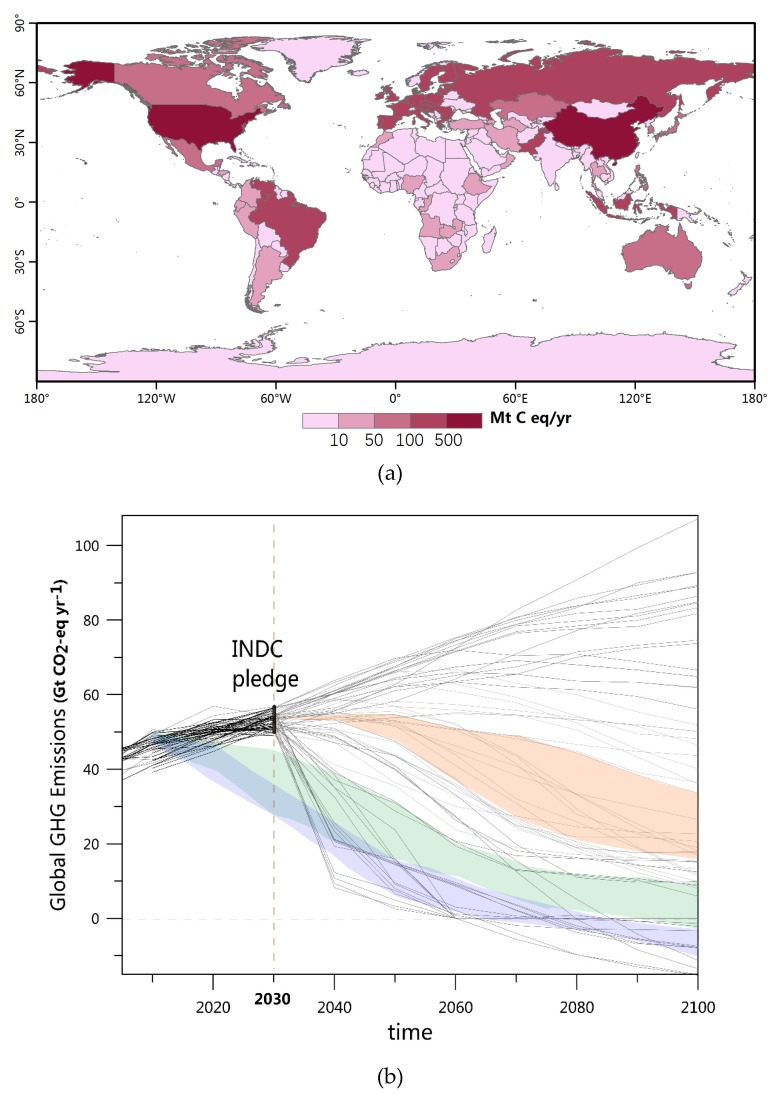
Global greenhouse gas (GHG) emissions of the Intended Nationally Determined Contributions (INDC) scenarios. (**a**) Emission reductions of countries with unconditional INDC pledges in 2030 (relative to the baseline development scenarios). (**b**) Global GHG emission pathways; grey lines indicate INDC pathways until the end of this century based on simulations of future emissions from 28 socioeconomic models. The different colours indicate ranges of INDC ‘continued action’ (orange), 2.0 °C (green), and 1.5 °C (blue) scenarios.

Published NDC provide the emission targets that are proposed to be achieved by 2030 (Figure 1a). To extend the INDC scenario to the end of this century, we assume that countries continue the mitigation efforts pledged in the INDC, and that the global emissions follow a relatively constant decarbonization rate after 2030. The simulation results of future emissions from 28 socioeconomic models (Intergovernmental Panel on Climate Change (IPCC) Fifth Assessment Report (AR5) scenario database, https://tntcat.iiasa.ac.at/AR5DB/) were analyzed considering factors such as the decarbonization rate, carbon capture and storage (CCS), energy structure innovation, and time of carbon neutralization. We selected scenarios in which the 2030 GHG emission level is consistent with the NDC (50–56 GtCO_2_eq/yr). Considering the difficulty and uncertainty of future CCS, scenarios with a CCS >15 GtCO_2_eq/yr were omitted. In this study, we focused on the NDC-sustained mitigation scenario (hereafter INDC) (Figure 1b), which is consistent with the ‘continued action’ pathway reported in Rogelj et al. (2016) [17], Wang et al. (2018) [20], and Climate Action Tracker (CAT) (2017) [21].

In addition, the 2.0 °C/1.5 °C temperature target scenarios consistent with the Paris Agreement were also analyzed in this study. The 2.0 °C/1.5 °C target scenarios are based on the Fifth Assessment Report (AR5) [2] and 1.5°C Special Report [22] of the Intergovernmental Panel on Climate Change (IPCC).

We estimated the global mean warming response to the NDC-sustained mitigation. Several studies provided evaluation results. For example, the United Nations Environment Program (UNEP) published the ‘Emissions Gap Report’ [19] and hypothesized that global emissions in accordance with the INDC development would result in a global warming of 3–4 °C (probability over 66%) above the preindustrial level by 2100, and would have a more profound impact on the climate. Rogelj et al. (2016) [17] showed that the median temperature rise at the end of this century will be ~2.6 °C to 3.1 °C, and a stronger temperature increase is likely. The CI (2016) [23] reported a range of ~2.0 °C to 4.6 °C based on INDC scenarios. Wang et al. (2018) [24] pointed out that the INDC emissions would lead to a global mean warming of 1.4 °C (1.3–1.7 °C) by 2030 and 3.2 °C (2.6–4.3 °C) by 2100. We synthesized the results from the above-mentioned studies and assessed the corresponding potential global mean temperature rise for various emission scenarios based on 78 climate sensitivity experiments from the GCM ensemble of CMIP5 [25]. After a comprehensive assessment, we determined $\Delta T_{INDC}\approx 2.9\sim 3.3\;{}^{\circ}C$ (median 3.1 °C) as the most likely range of temperature increase for the “continued action” pathways of INDCs.

### 2.2. Simulation of the Climate Models

We used daily minimum and maximum near-surface air temperatures data of 32 GCMs from CMIP5 [25]. Basic information about these models is provided in Table S1. Since the ensemble mean of these GCM models can filter the uncertainty due to the inter-model variability, it is the best representation of the response to the imposed external forcing. Its predictions are better than those of any individual member [26,27]; therefore, it was used to reflect the simulated extreme high-temperature changes in this study. We selected only the first run for the multi-ensemble models so that all the models were treated equally. All the model data were interpolated to a common 1° × 1° horizontal grid using a bilinear interpolation algorithm for ensemble analysis.

The period of 1985–2005 is referred to as the present-day period. The preindustrial period in this study is 1861–1900. The CA includes Kazakhstan (KAZ), Uzbekistan (UZB), Turkmenistan (TKM), Kyrgyzstan (KGZ), and Tajikistan (TJK).

Based on previous research [28,29,30], we used a time-slice approach based on which the spatial state at a specific warming point related to $\Delta T_{INDC}$ (or 2.0 °C, 1.5 °C) is taken from decadal time slices with the respective mean warming for each model separately. In detail, the time series of the globally averaged surface air temperature was smoothed using a 21-year running mean to filter out the interannual variability of the individual model. The used 21-year time slice includes the median year during which the global mean surface temperature exceeded a given warming target ($\Delta T_{INDC}$, 1.5 °C, or 2.0 °C; see Table S2), the 10 years preceding, and the following median year. This method can also be applied for other climate indicators, and thus can relate various indicators to mean global warming.

### 2.3. Probability Ratio

Many investigations indicated that the probability density curves of the SAT shift to the right side with increasing global warming, indicating an increase in the mean SAT associated with more frequent occurrences of very hot and extremely hot weather. The shape of the curves widens, suggesting the enlargement of the standard deviation of the SAT [2]. We calculated the probability ratio (PR) for temperatures with different intensities between two scenarios to quantify this change using the following equation:

(1) $$\mathrm{PR}=\frac{P_{1}}{P_{0}},$$

where PR is the probability ratio, $P_{0}$ is the probability of a specific temperature intensity in the present climatology (e.g., the probability of a temperature higher than the 80th percentile and lower than the 90th percentile is 10%, and the present-day PR for this temperature interval is 1.0), and $P_{1}$ is the corresponding probability of reaching this temperature intensity in future scenarios.

### 2.4. Extreme High-Temperature Indices

High-temperature events can be defined using either relative or absolute thresholds. The six extreme high-temperature indices that were applied in this study are listed in Table 1. The indices three-day warm day event (TMX3day) and three-day warm night event (TNX3day) were used as representatives of the indices defined according to the intensity, denoting several continuous days/nights during which extremely warm temperatures are prevalent. There is no universal and rigorous definition of heat wave (HW) when considering high-temperature events despite various studies, because the research regions and methods applied in the past considerably vary [31]. In this study, a HW index was defined as a consecutive period of at least three days during which the daily maximum temperature exceeded the 95th percentile of the baseline period. The percentile threshold was ≥30 °C, and HW represents the impact of the longest period of hot days. The advantage of the combination of relative and absolute thresholds is the wide applicability to diverse climatological regions [32].

In addition, other widely used extreme temperature indices include a duration index (warm spell duration index, WSDI) and two frequency indices (warm days, TX90p, and warm nights, TN90p) [33]. When calculating the percentile-based indices, a bootstrap procedure recommended by Zhang et al. (2005) [34] was used for the baseline period (1961–1990) to avoid inhomogeneity across the in-base and out-base periods.

Regarding social impacts, the projected population distributions under different shared socioeconomic pathways (SSPs) [35] were used to investigate the population-weighted changes of the extreme high-temperature indices. These SSPs describe five alternative outcomes for trends in demographics, economics, technological development, lifestyles, governance, and other societal factors. Projections of spatial population change are quantitatively consistent with national population and urbanization projections for the SSPs and qualitatively consistent with assumptions in the SSP narratives regarding spatial development patterns. These projections are produced by a parameterized gravity-based downscaling model. In this paper, we only show the population-related results estimated from the population projected for the year 2100 under the SSP2 scenario. However, the population exposures based on projections under other SSP scenarios are qualitatively similar.

The Wilcoxon rank sum test was applied to identify if there is a statistical significance of differences between two warming levels based on multi-model results.

### 2.5. Avoided Impacts

The impacts of extreme high-temperature events that are avoided by a lower warming level (e.g., 2.0 °C or 1.5 °C) compared with the INDC scenario were investigated using the following equation:

(2) $$\mathrm{Avoid}\;\mathrm{Imapct}=\frac{C_{INDC}-C_{k}}{C_{INDC}},$$

where C represents the changes in extreme high-temperature indices under a specific scenario compared with the present climatology, and the subscript k indicates different scenarios (e.g., 2.0 °C/1.5 °C). Such information is beneficial for future mitigation and adaptation planning.

## 3. Results

### 3.1. Changes in the Surface Air Temperature

**Figure 2:**
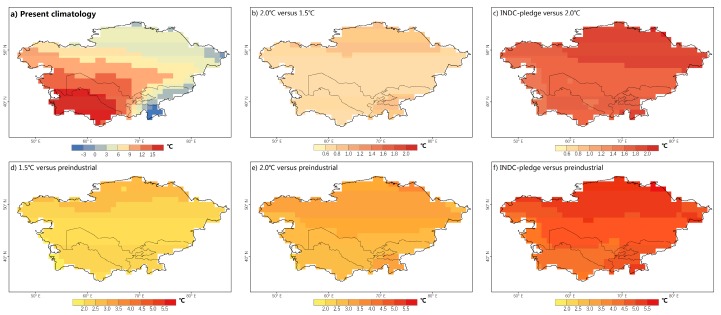
(**a**) Present annual mean temperature over Central Asia; (**b–f**) Changes in the annual mean temperature over Central Asia. The differences between different sets of scenarios are displayed in the top-left corner (based on the multi-model ensemble mean).

CA will experience a remarkably higher warming rate than the global mean based on various scenarios. The regional mean SAT in CA will be 2.3 °C (1.8–2.7 °C, 25–75% interval range), 3.0 °C (2.5–3.5 °C), and 4.8 °C (4.2–5.3 °C) above the preindustrial level under the 1.5 °C, 2.0 °C, and INDC scenarios, respectively. With respect to the spatial pattern, the warming will be stronger in high-latitude areas and in the Pamir Mountains in all scenarios. The differences between the INDC/2.0 °C (or 2.0 °C/1.5 °C) scenarios are also larger in these areas (Figure 2).

**Figure 3:**
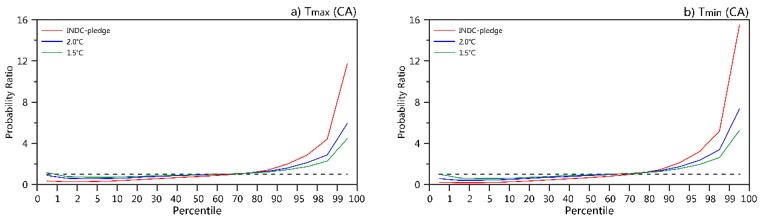
Regional mean probability ratio (PR) values over Central Asia for the responses of the daily maximum (**a**) and minimum (**b**) temperatures to the 1.5 °C, 2.0 °C, and $\Delta T_{INDC}$ global warming levels based on the percentile thresholds determined by the 1985–2005 climatology based on the multi-model ensemble mean. The dashed line represents a value of 1.0. The regional mean PR values for five subregions refer to Figure S2.

We further analyzed the changes in the regional mean daily maximum and minimum SAT structures of the three scenarios compared with the present climatology (Figure 3). The high-temperature PR (e.g., higher than the 80th percentile of the present climatology) increases, while the low-temperature PR (e.g., lower than the 20th percentile of the present climatology) decreases. The extreme high-temperature PR increases much more than the mild high-temperature PR. With respect to the daily maximum temperatures higher than the 99th percentile of the present day in CA, the PR will be 4.5, 5.9, and 11.7 times that of the present day in the 1.5 °C, 2.0 °C, and INDC scenarios, respectively. The increasing PR amplitudes of nighttime hot extremes are larger than those of daytime hot extremes. Similarly, the PR of the daily maximum temperatures higher than the 99th percentile of the present day in CA will be 5.3, 7.4, and 15.5, respectively. Among the countries in CA, the increases in the PRs of extremely high temperatures are strong in TJK and KGZ (Figure S2).

### 3.2. Changes in Extreme High-Temperature Events

**Figure 4:**
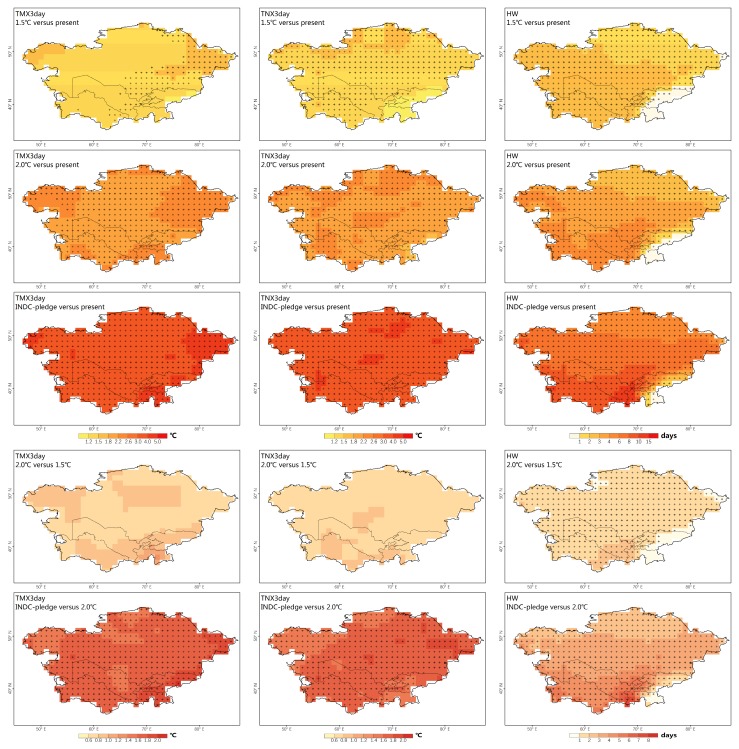
Changes in TMX3day (column I), TNX3day (column II), and HW (column III) over Central Asia, based on the multi-model ensemble mean. The differences between different sets of scenarios are displayed in the top-left corner. The dotted areas are statistically significant at the 5% level according to Wilcoxon’s rank-sum test.

**Figure 5:**
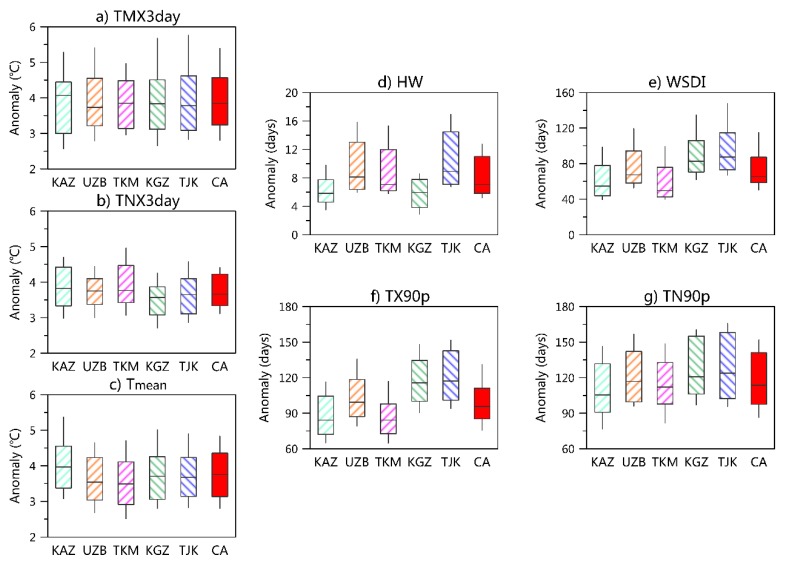
Population density-weighted average differences among the INDC-pledge scenario and the present level of the (**a**) TMX3day, (**b**) TNx3day, (**c**) annual mean temperature, (**d**) HW, (**e**) WSDI, (**f**) TX90p, and (**g**) TN90p in Central Asia and its subregions. The box-whisker plots show the multi-model ensemble 10th, 25th, 50th, 75th, and 90th intervals. The population density-weighted average was estimated based on the population prediction for 2100 under the socioeconomic pathway 2 (SSP2) scenario. The results of regional average refer to Figure S3. KAZ = Kazakhstan; UZB = Uzbekistan, TKM = Turkmenistan, KGZ = Kyrgyzstan, and TJK = Tajikistan.

We first focused on the changes in the intensity index (i.e., TMX3day and TNX3day). The increasing amplitude of the TMX3day and TNX3day are relatively uniform across CA under the three scenarios (Figure 4). Considering the population density-weighted change, the increasing amplitudes of the TMX3day and TNX3day are in general relatively close to those of the annual mean SAT (Figure 5). In most countries, the impact of TMX3day is slightly more pronounced than that of TNX3day.

**Figure 6:**
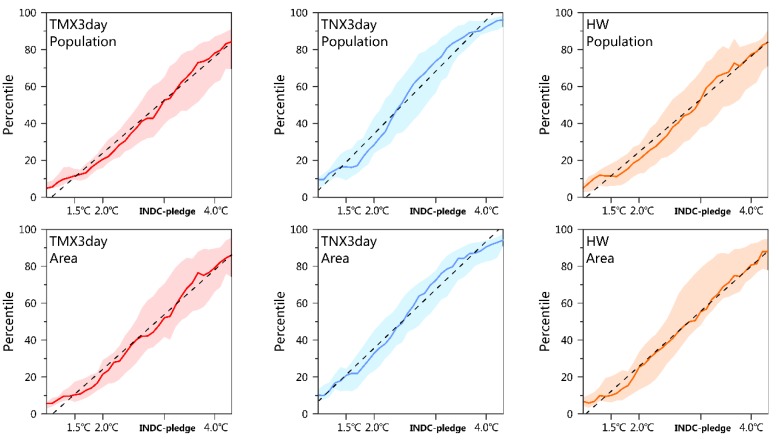
The fraction of the population and area in Central Asia for which the historical TMX3day (column I), TNX3day (column II), and HW (column III) record defined from 1961–2005 is broken under different global warming levels. The multi-model medians are the solid lines and the interquartile ranges are shaded. The dashed black lines denote the linear trend of the population/area fraction with the global mean warming. Fractions of the population and area in five subregions refer to Figure S4.

Record-breaking high-temperature events will be more frequent. To estimate the scope of the impact of record-breaking events under different scenarios, we first calculated the fraction of the population/area in CA for which the TNX3day record defined during the historical period (1961–2005) was broken in any given year, and then smoothed it by the 21-year running mean. Approximately 51%/52% (72%/71%) of the population/area in CA will break the historical TMX3day (TNX3day) record under the INDC scenario, respectively (Figure 6). A comparison of regional changes reveals that the TKM and TJK will experience stronger changes (Figure S4). An approximately linear increase in the population/area fraction with the global mean warming can be observed in all regions, and the increasing rates of record-breaking nighttime hot extremes (i.e., TNX3day) are larger than those of daytime hot extremes (i.e., TMX3day).

In contrast to the uniform changes in TMX3day and TNX3day, the change magnitudes of HWs show regional differences, with larger magnitudes in the low-altitude areas (Figure 4). A notable increase lasting more than seven days mainly occurs in the south of CA (except for the Pamir Mountains) under the INDC scenario. The population density-weighted average changes are more intense in UZB, TKM, and TJK (Figure 5). The spatial patterns are similar, but the changes in the HWs under the 2.0°C/1.5°C scenarios are less significant compared with the INDC scenario (Figure 4). Record-breaking HW events will also remarkably increase (similar to TMX3day and TNX3day); approximately 55%/51% of the population/area in CA will break the historical record under the INDC-pledge scenario. The increased amount of record-breaking HW events will be more evident in TKM and UZB.

In this section, the changes of other extreme indices are considered. For extreme indices defined according to frequency, the changes of the daytime extremes (i.e., TX90p) are smaller than those of the nighttime extremes (i.e., TN90p) (Figure 5). The spatial patterns of three percentile-based indices (TX90p, TN90p, and WSDI) are similar; that is, more intense increases are observed in the south than in the north (Figure S5).

### 3.3. Impacts Avoided Based on Low Warming Scenarios

**Figure 7:**
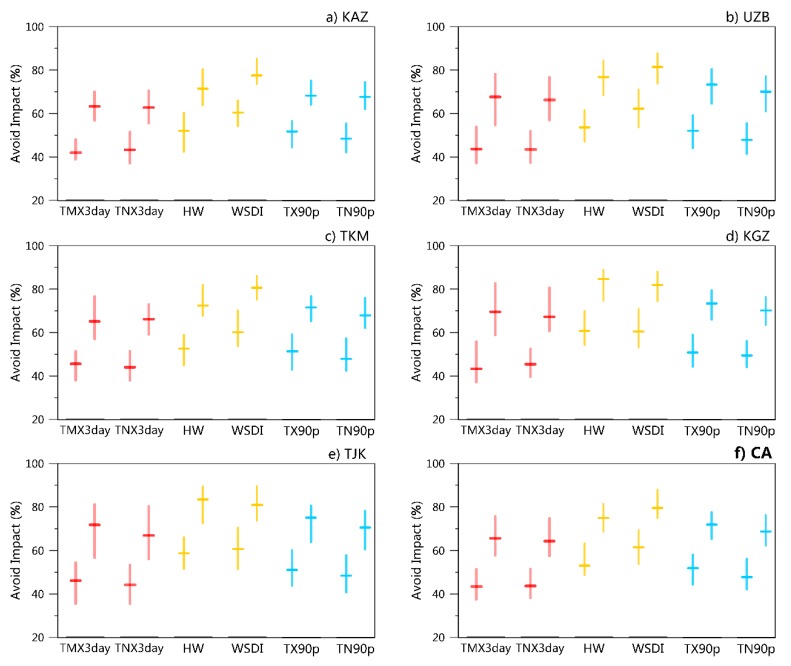
Changes of extreme high-temperature events avoided over Central Asia and its subregions in less warming scenarios (units: %). Population density weighted average extreme temperature indices is reduced in the low warming scenarios (left—2.0 °C compared to the INDC pledge, right—1.5 °C compare to INDC pledge). The red boxes represent the indices defined by intensity, the yellow boxes represent the indices defined by duration, and the blue boxes represent the indices defined by frequency. Central lines and bars denote multimodal medians and interquartile ranges, respectively. For the avoid impact based on regional average extreme temperature indices, refer to Figure S6.

If the warming is limited to a lower level, the CA region is projected to benefit from robust reductions in extreme high-temperature events (Figure 7). Compared with the INDC scenario, the lower warming of the 2.0 °C/1.5 °C target scenarios will help to avoid approximately 44%/65% of the increase in the intensity of extreme high-temperature events (TMX3day and TNX3day), 53–61%/75–80% of the increase in the duration of extreme high-temperature events (HW and WSDI), and approximately 48–52%/69–72% of the increase in the frequency of extreme high-temperature events (TX90p and TN90p) in CA based on the population density-weighted average indices. All the subregions would experience such a significant impact reduction, although the magnitudes would slightly differ.

## 4. Discussion

Climate warming in response to actual emission reductions within the framework of the Paris Agreement remains an ongoing interest. Most of the published studies are based on representative concentration pathways (RCP) or 2.0 °C/1.5 °C scenarios, which do not account for the current national mitigation commitments negotiated by governments. In this study, we quantified the regional climate change based on the self-determined emission reduction commitments made in climate negotiations as the starting point to assess the future climate response. Our results indicate that the climate warming under the INDC scenarios is projected to greatly exceed the long-term goal of the Paris Agreement of stabilizing the global mean temperature above the 2.0 °C or 1.5 °C level. Extreme high-temperature events in CA will become more intense, more frequent, and longer-lasting with the enhancement of global warming, and their responses to global warming are basically linear. If the global emission reductions are further strengthened to achieve the ambitious 2.0 °C/1.5 °C temperature target, the benefits with respect to the reduction of the regional risk associated with record-breaking high-temperature events are remarkable.

The definition of a HW (extreme high-temperature event) has a direct impact on the estimations of the HW intensity, frequency, trend, and spatial distribution [31]. The slight differences in the projected HWs between different studies could be explained by the definition of the HW and/or the dataset used. Furthermore, HWs should be defined based on a combination of temperature and humidity, which are more closely associated with human health [36,37,38]. When discussing the impact of extreme high-temperature events, non-meteorological components such as the human fitness and activity level, and physiological adaptation to an environment are important influencing factors [39]. Our study does not discuss the vulnerability of socioeconomic systems; the results reflect only the risk of physical climate change.

Previous studies, which are based on the CMIP5 dataset, were not specifically designed for the assessment of the climate response under the Paris Agreement. The recent NCAR CESM Low-Warming [40] and HAPPI experiments [41], which aim to quantify the impacts on weather-related risks corresponding to a warming of 1.5 °C or 2.0 °C, are not specific to the INDC pledges, which are the focus of this study. Currently, the targeted experimental design concerning INDC emissions that are consistent with the Paris Agreement is still lacking. In addition, the resolutions of GCMs are too coarse to more precisely capture regional signals of climate extremes, while regional climate models (RCMs) with finer resolutions and more sophisticated processes have better abilities to reproduce the regional climate characteristics [42]. Thus, a targeted experiment associated with INDC emission pledges based on RCMs is needed to better project the changes in extreme high-temperatures at smaller regional scales in CA and provide policy makers with more precise information.

## 5. Conclusions

Based on simulations from 32 GCMs models and their ensemble, we examined changes in extreme high-temperature events in CA under global INDC scenarios and further compared the results with those of the 2.0 °C/1.5 °C warming targets. Our primary conclusions are as follows:

(1) The SAT in CA will increase by approximately 2.3 °C, 3.0 °C, and 4.8 °C above the preindustrial level under the 1.5 °C, 2.0 °C, and INDC scenarios, with a higher warming rate than the global mean. Larger warming magnitudes will occur in high-latitude areas and the Pamir Mountains. The amplitudes of the PRs of extreme high temperatures will increase much more than those of the mild high temperatures.
(2) Extreme high-temperature events will become more intense, more frequent, and longer-lasting with the enhancement of global warming. The increasing amplitude of the intensity is relatively uniform among CA, while the duration of HWs will increase more in low-altitude areas. The nighttime heat extremes will increase more than the daytime hot extremes (with respect to the frequency index and PR). Record-breaking high-temperature events will be more frequent, and the population/area fraction will linearly increase with the global mean warming.
(3) Compared with the INDC scenario, the lower warming of the 2.0 °C/1.5 °C target scenarios will help to avoid approximately 44–61%/65–80% of the increase in extreme temperature events in terms of the intensity, frequency, and duration in CA. All the subregions would experience such a remarkable impact reduction, although the magnitudes would slightly differ.

## Figures and Tables

**Table 1:** Definitions of extreme high-temperature indices used in this study (present climatology of these extreme high-temperature indices refers to Figure S1).

Name	Label	Definition (Unit)
Three-day warm day event	TMX3day	The highest three-day mean daily maximum temperature in a year (°C)
Three-day warm night event	TNX3day	The highest three-day mean daily minimum temperature in a year (°C)
Heat wave duration index	HW	The longest consecutive period of at least three days during which the daily maximum temperature exceeded the 95th percentile of the base period of 1961–1990 and the percentile threshold was ≥30 °C (days). The specified percentile of the base period is calculated on moving daily data with a five-day window, similarly hereinafter in this table.
Warm spell duration index	WSDI	Annual number of days with at least six consecutive days during which the daily maximum temperature (TX) >90th percentile (days)
Warm days	TX90p	Let TX_ij_ be the daily maximum temperature on day i in period j, and let TX_in_90 be the calendar day 90th percentile centered on a five-day window for the base period of 1961–1990. The number of days is determined during which TX_ij_ > TX_in_90 (days)
Warm nights	TN90p	Similar to TX90p, but for the daily minimum temperature (days)

## Supplementary Materials

The following are available online at https://www.mdpi.com/1660-4601/16/15/2661/s1, Table S1: Basic information of 32 GCMs in CMIP5, Table S2: Time period for the warming in global surface temperature relative to preindustrial era reaching the threshold as indicated by individual models, Figure S1: Present climatology of (a) TMX3day, (b) TNX3day, (c) HW, (d) WSDI, (e) TX90p, and (f) TN90p over central Asia, based on multi-model ensemble mean, Figure S2: Regional mean probability ratio (PR) values over five countries for the responses of daily maximum (a) and minimum (b) temperatures to the 1.5 °C, 2.0 °C, and $\Delta T_{INDC}$ global warming level based on the percentile thresholds determined by the 1985–2005 present climatology, based on the multi-model ensemble mean. The dashed line represents value of 1.0, Figure S3: Regional average differences among INDC-pledge scenario and present level in (a) TMX3day, (b) TNx3day, (c) annual mean temperature, (d) HW, (e) WSDI, (f) TX90p, and (g) TN90p in Central Asia and the five countries. The box-whisker plots show the multi-model ensemble’s 10th, 25th, 50th, 75th, and 90th intervals, Figure S4: The fraction of population and area in five countries where the historical TMX3day, TNX3day, and HW record defined during 1961–2005 is broken in different global warming levels. The multi-model medians are in solid lines, and interquartile ranges are shaded. The dashed black lines denote the linear trend of population/area fraction with global mean warming, Figure S5: Changes in TX90p (column I), TN90p (column II), and WSDI (column III) over Central Asia, based on multi-model ensemble mean. The differences between different sets of scenarios are labeled on the top-left. The dotted areas are statistically significant at the 5% level according to Wilcoxon’s rank-sum test, Figure S6: Changes of extreme high-temperature events avoided over Central Asia and its subregions in less warming scenarios (units: percentage). Regional average extreme temperature indices are reduced in the low warming scenarios (left—2.0°C compared to INDC pledge, right—1.5°C compare to INDC pledge). The red boxes represent the indices defined by intensity, the yellow boxes represent the indices defined by duration, and the blue boxes represent the indices defined by frequency. Central lines and bars denote multimodal medians and interquartile ranges, respectively.
