# Supplementary material for: Regional Temperature Response in Central Asia to National Committed Emission Reductions
Source: Int J Environ Res Public Health. 2019 Jul 25;16(15):2661. doi: 10.3390/ijerph16152661 (PMC6696578; doi:10.3390/ijerph16152661)
Supplement: Supplementary file 1 [file ijerph-16-02661-s001.pdf]

Table S1. Basic information of 32 GCMs in CMIP5.

Model name	Institute	Country	Horizontal resolution
**ACCESS1-0**	Commonwealth Scientific and Industrial Research Organization/Bureau of Meteorology	Australia	192×145
**ACCESS1-3**			192×145
**BCC-CSM1-1**	Beijing Climate Center, China Meteorological Administration	China	128×64
**BCC-CSM1-1-m**			320×160
**BNU-ESM**	Beijing Normal University	China	128×64
**CanESM2**	Canadian Centre for Climate Modelling and Analysis	Canada	128×64
**CCSM4**	National Center for Atmospheric Research	USA	288×192
**CESM1-BGC**			288×192
**CMCC-CESM**	Centro Euro-Mediterraneo sui Cambiamenti Climatici	Italy	96×96
**CMCC-CM**			480×240
**CMCC-CMS**			192×96
**CNRM-CM5**	Centre National de Recherches Météorologiques, Centre Européen de Recherche et de Formation Avancée en Calcul Scientifique	France	256×128
**CSIRO-Mk3-6-0**	Commonwealth Scientific and Industrial Research Organization/ Queensland Climate Change Centre of Excellence	Australia	192×96
**EC_EARTH**	EC-EARTH consortium	Netherland s/Iceland	128×60
**FGOALS-s2**	Institute of Atmospheric Physics, Chinese Academy of science	China	128×108
**GFDL-CM3**	Geophysical Fluid Dynamics Laboratory	USA	144×90
**GFDL-ESM2G**			144×90
**GFDL-ESM2M**			144×90
**HadGEM2-CC**	Met Office Hadley Centre	UK	192×145
**HadGEM2-ES**			192×145
**INM-CM4**	Russian Academy of Sciences, Institute of Numerical Mathematics	Russia	180×120
**IPSL-CM5A-LR**	Institut Pierre Simon Laplace	France	96×96
**IPSL-CM5A-MR**			144×143
**IPSL-CM5B-LR**			96×96
**MIROC5**	Atmosphere and Ocean Research Institute (The University of Tokyo), National Institute for Environmental Studies, and Japan Agency for Marine-Earth Science and Technology	Japan	256×128
**MIROC-ESM**			128×64
**MIROC-ESM-CHEM**			128×64
**MPI-ESM-LR**	Max Planck Institute for Meteorology	Germany	192×96
**MPI-ESM-MR**			192×96
**MRI-CGCM3**	Meteorological Research Institute	Japan	320×160
**MRI-ESM1**			320×160
**NorESM1-M**	Bjerknes Centre for Climate Research, Norwegian Meteorological Institute	Norway	144×96

Table S2. Time period for the warming in global surface temperature relative to preindustrial era reaching the threshold as indicated by individual models.

**Model**	1.5℃	2.0℃	ΔT_{INDC}	**Model**	**1.5**℃	**2.0**℃	ΔT_{INDC}
ACCESS1-0	2027	2040	2062	GFDL-ESM2G	2037	2054	2082
ACCESS1-3	2031	2042	2063	GFDL-ESM2M	2036	2052	2085
BCC-CSM1-1	2021	2037	2062	HadGEM2-CC	2028	2041	2059
BCC-CSM-1-1-m	2014	2031	2063	HadGEM2-ES	2024	2037	2057
BNU-ESM	2009	2023	2047	INM-CM4	2045	2058	2088
CanESM2	2013	2027	2051	IPSL-CM5A-LR	2010	2026	2049
CCSM4	2015	2030	2060	IPSL-CM5A-MR	2016	2031	2052
CESM1-BGC	2018	2034	2062	IPSL-CM5B-LR	2023	2038	2065
CMCC-CESM	2038	2047	2070	MIROC5	2033	2049	2074
CMCC-CM	2030	2042	2063	MIROC-ESM	2021	2030	2055
CMCC-CMS	2031	2042	2064	MIROC-ESM-CHEM	2019	2031	2052
CNRM-CM5	2031	2045	2070	MPI-ESM-LR	2017	2036	2063
CSIRO-Mk3-6-0	2034	2045	2068	MPI-ESM-MR	2020	2039	2062
EC-EARTH	2019	2035	2063	MRI-CGCM3	2041	2053	2079
FGOALS-s2	1998	2012	2040	MRI-ESM1	2037	2050	2075
GFDL-CM3	2023	2035	2056	NorESM1-M	2033	2050	2076

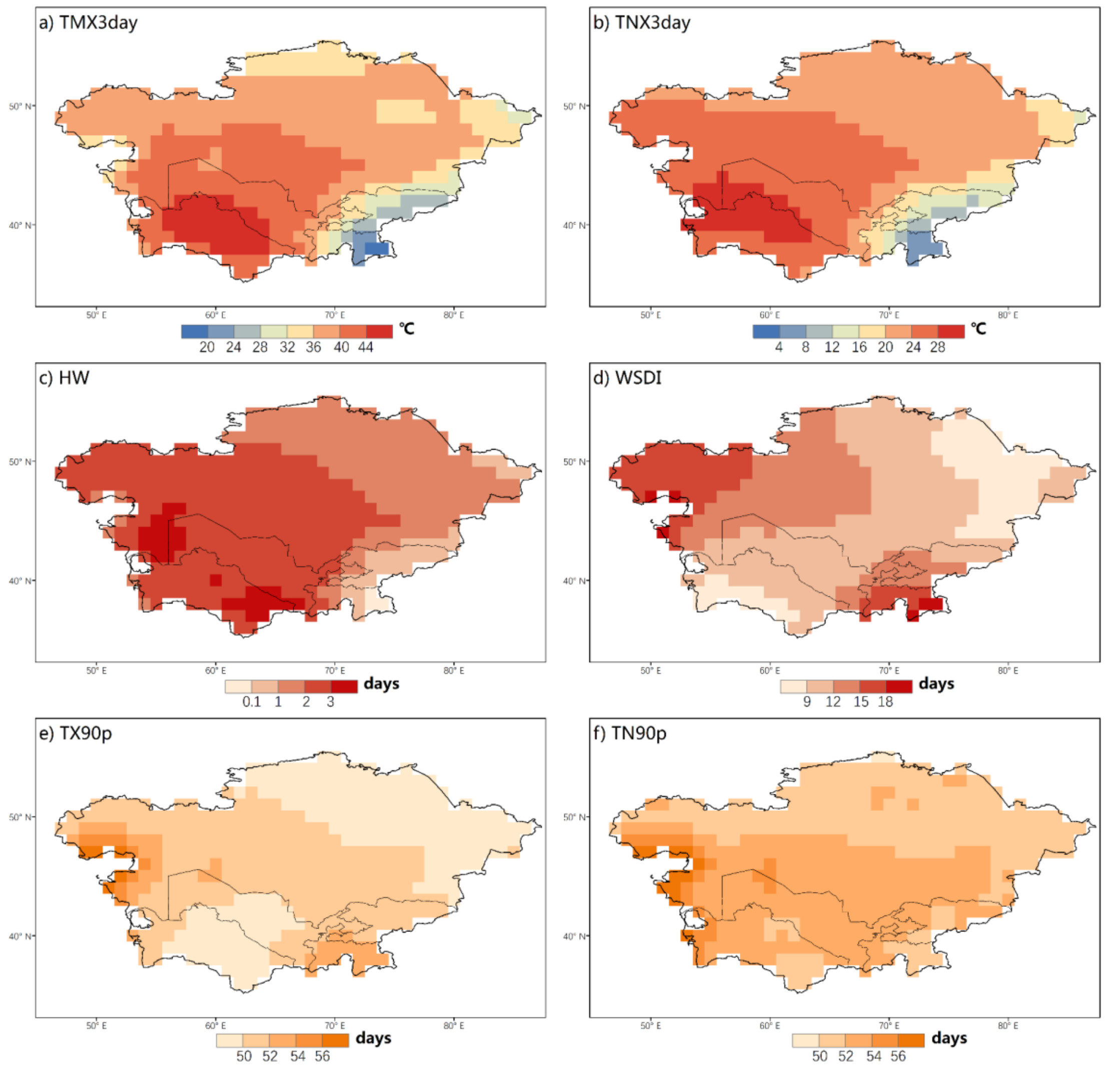

Figure S1 Present climatology of (a) TMX3day, (b) TNX3day, (c) HW, (d) WSDI, (e) TX90p, and (f) TN90p over central Asia, based on multi-model ensemble mean.

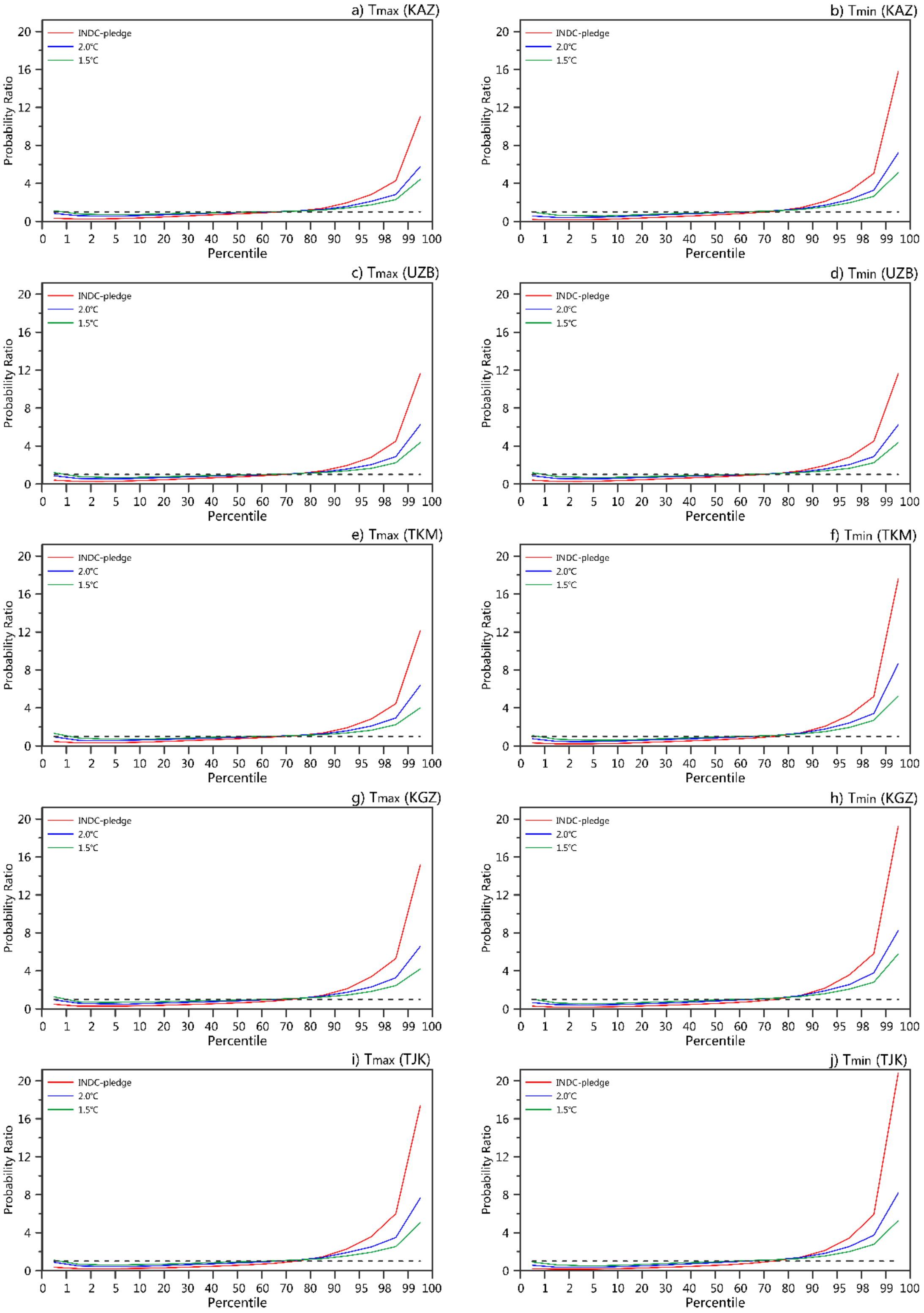

Figure S2. Regional mean probability ratio (PR) values over five countries for the responses of daily maximum **(a)** and minimum **(b)** temperatures to the 1.5°C, 2.0°C and ΔT_{INDC} global warming level based on the percentile thresholds determined by the 1985–2005 present climatology, based on multi-model ensemble mean. The dashed line represents value of 1.0.

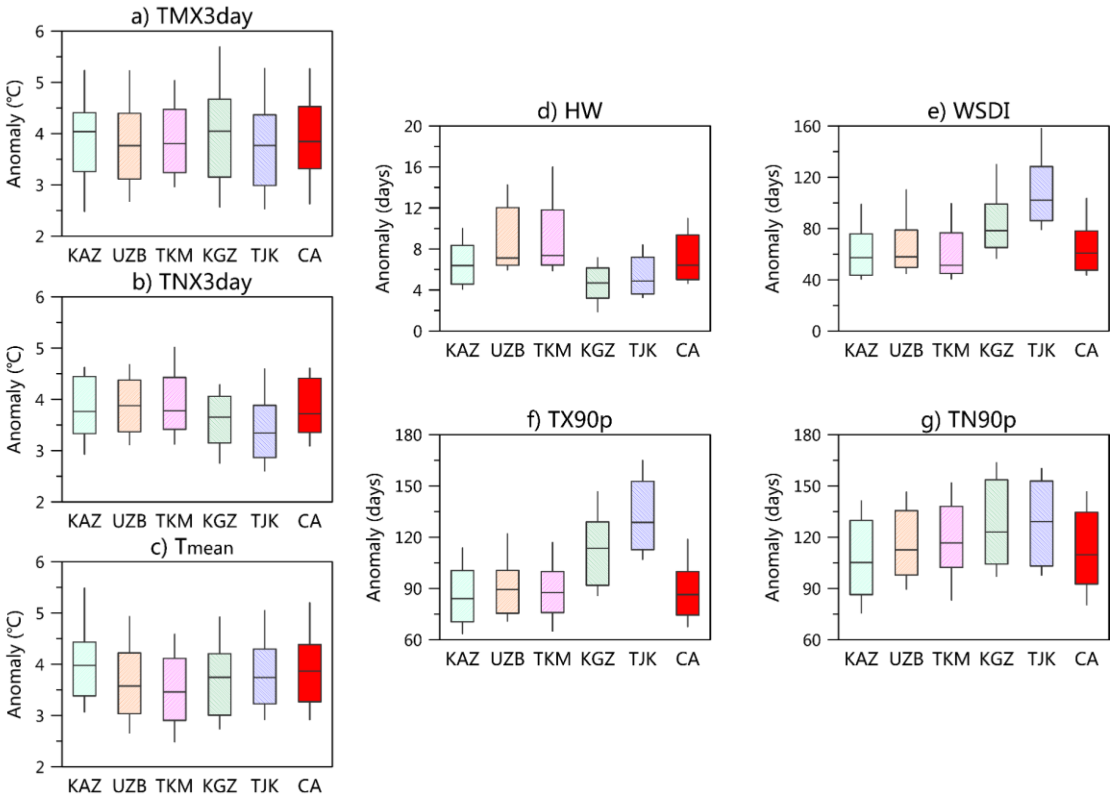

Figure S3 Regional average differences among INDC-pledge scenario and present level in **(a)**TMX3day, **(b)** TNx3day, **(c)** annual mean temperature, **(d)** HW, **(e)** WSDI, **(f)** TX90p, and **(g)** TN90p in Central Asia and the five countries. The box-whisker plots show the multi-model ensemble 10th, 25th, 50th, 75th, and 90th intervals.

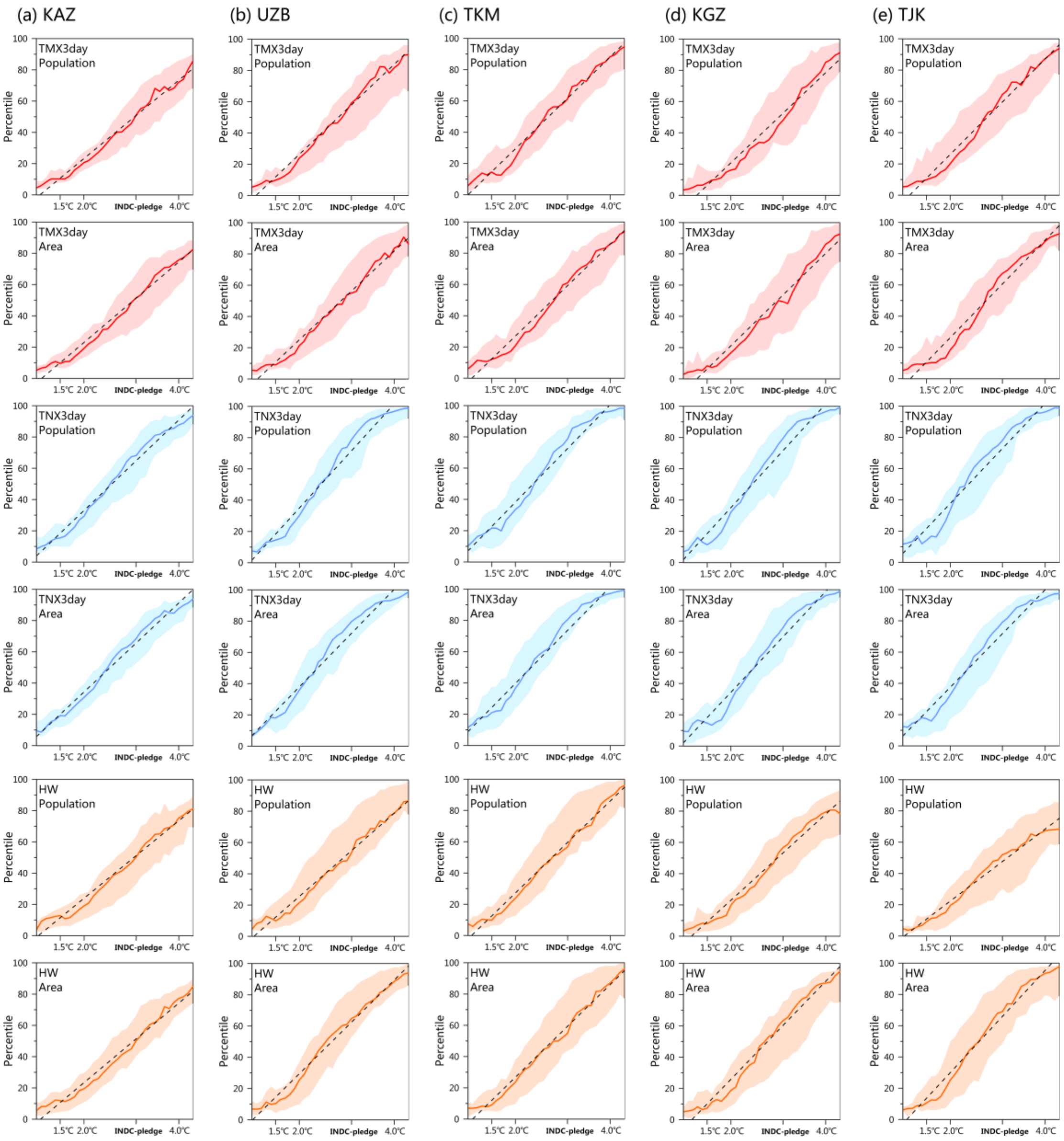

Figure S4 The fraction of population and area in five countries where the historical TMX3day, TNX3day, and HW record defined during 1961–2005 is broken in different global warming levels. The multi-model medians are in solid lines, and interquartile ranges are shaded. The dashed black lines denote the linear trend of population/area fraction with global mean warming.

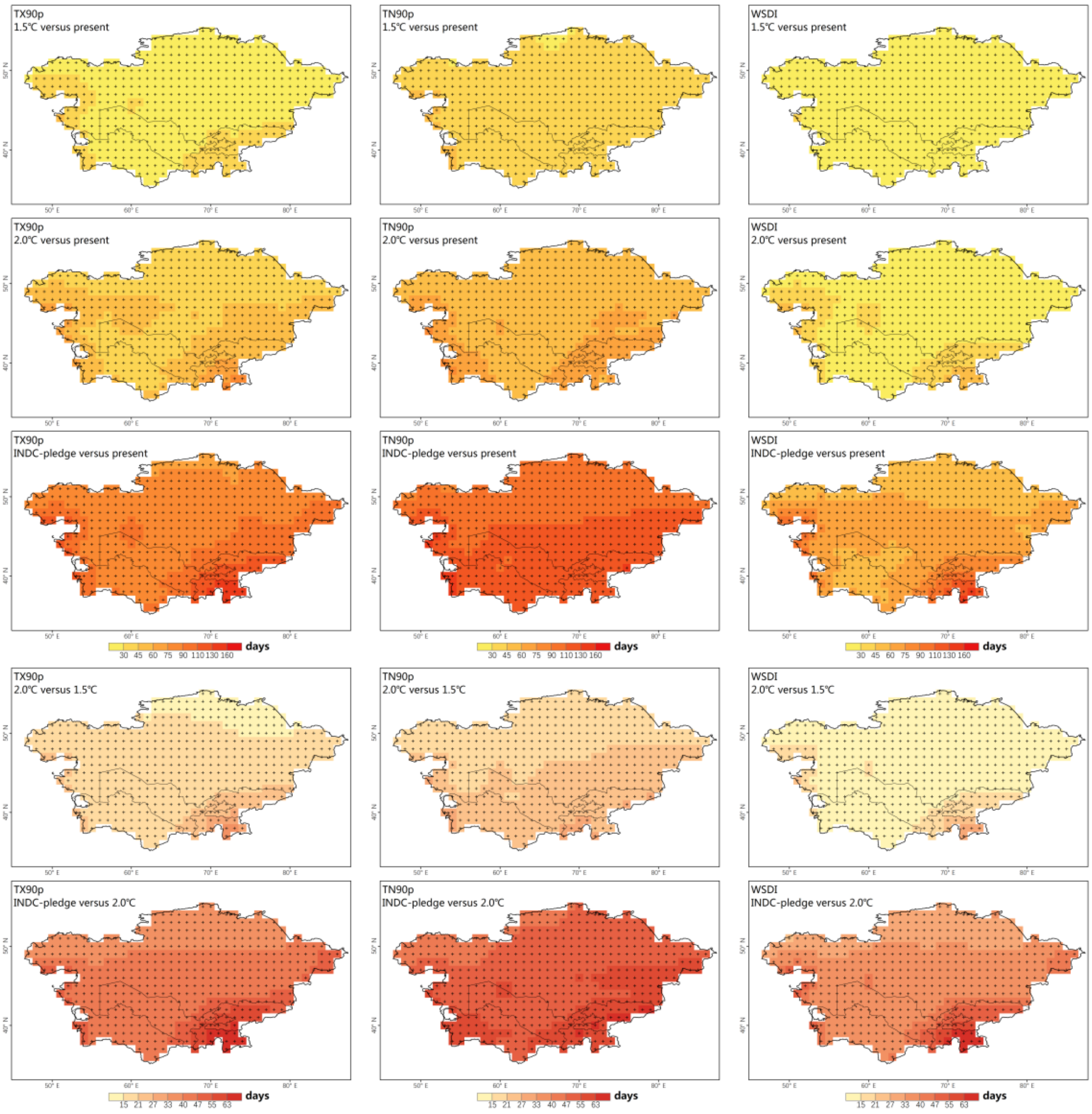

Figure S5 Changes in TX90p (column I), TN90p (column II), and WSDI (column III) over Central Asia, based on multi-model ensemble mean. The differences between different sets of scenarios are labelled on the top-left. The dotted areas are statistically significant at the 5% level according to Wilcoxon's rank-sum test.

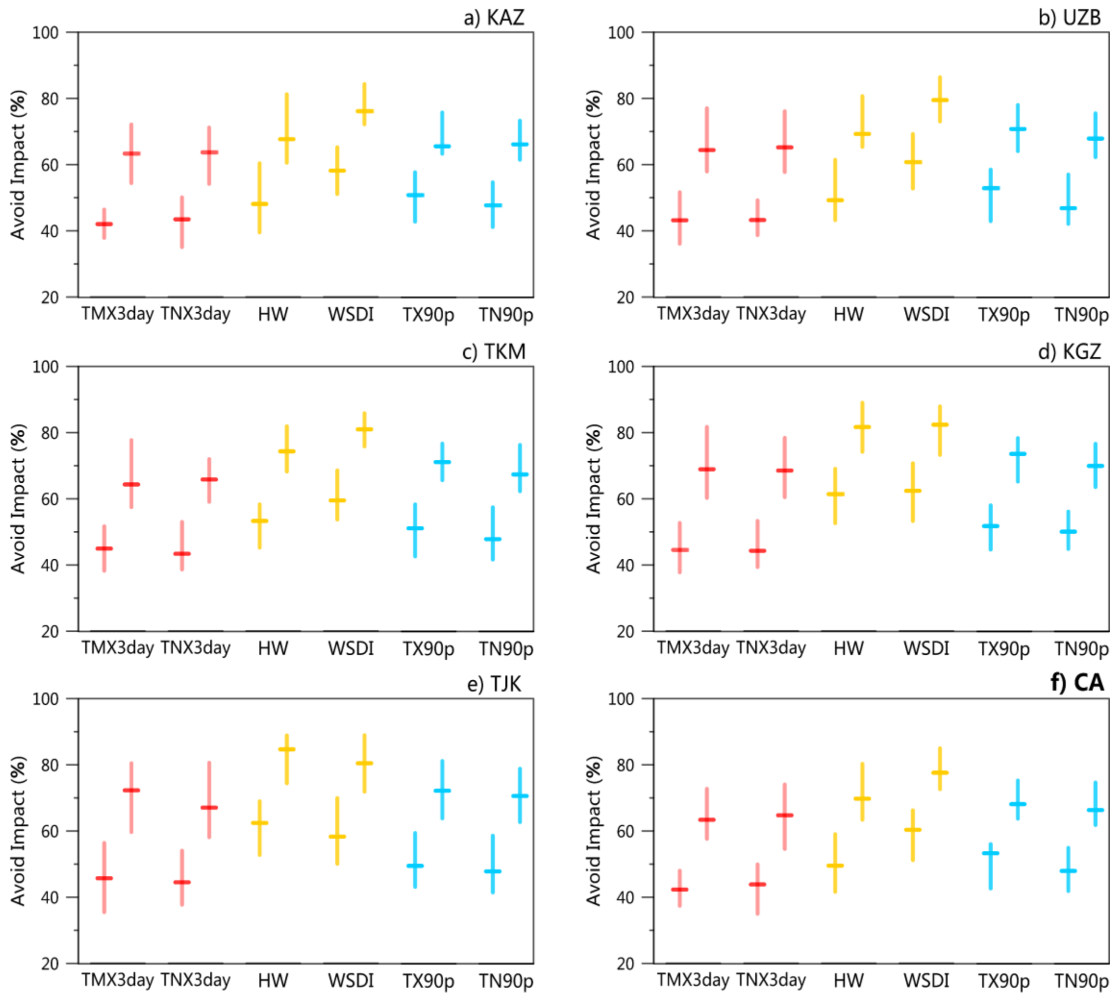

Figure S6 Changes of extreme high-temperature events avoided over Central Asia and its subregions in less warming scenarios (units: %). Regional average extreme temperature indices are reduced in the low warming scenarios (left – 2.0°C compared to INDC pledge, right – 1.5°C compare to INDC pledge). The red boxes represent the indices defined by intensity, the yellow boxes represent the indices defined by duration, and the blue boxes represent the indices defined by frequency. Central lines and bars denote multimodal medians and interquartile ranges, respectively.
